# Evaluation of copy number variation detection for a SNP array platform

**DOI:** 10.1186/1471-2105-15-50

**Published:** 2014-02-21

**Authors:** Xin Zhang, Renqian Du, Shilin Li, Feng Zhang, Li Jin, Hongyan Wang

**Affiliations:** 1 State Key Laboratory of Genetic Engineering and MOE Key Laboratory of Contemporary Anthropology, School of Life Sciences, Fudan University, 220 Handan Road, Shanghai 200433, China; 2 Children’s Hospital of Fudan University, 399 Wanyuan Road, Shanghai 201102, China

**Keywords:** CNV, CGH, Evaluation, Comparison, Performance test, Reproducibility test, Success rate, Birdsuite, dChip, GTC, PennCNV

## Abstract

**Background:**

Copy Number Variations (CNVs) are usually inferred from Single Nucleotide Polymorphism (SNP) arrays by use of some software packages based on given algorithms. However, there is no clear understanding of the performance of these software packages; it is therefore difficult to select one or several software packages for CNV detection based on the SNP array platform.

We selected four publicly available software packages designed for CNV calling from an Affymetrix SNP array, including Birdsuite, dChip, Genotyping Console (GTC) and PennCNV. The publicly available dataset generated by Array-based Comparative Genomic Hybridization (CGH), with a resolution of 24 million probes per sample, was considered to be the “gold standard”. Compared with the CGH-based dataset, the success rate, average stability rate, sensitivity, consistence and reproducibility of these four software packages were assessed compared with the “gold standard”. Specially, we also compared the efficiency of detecting CNVs simultaneously by two, three and all of the software packages with that by a single software package.

**Results:**

**S**imply from the quantity of the detected CNVs, Birdsuite detected the most while GTC detected the least. We found that Birdsuite and dChip had obvious detecting bias. And GTC seemed to be inferior because of the least amount of CNVs it detected. Thereafter we investigated the detection consistency produced by one certain software package and the rest three software suits. We found that the consistency of dChip was the lowest while GTC was the highest. Compared with the CNVs detecting result of CGH, in the matching group, GTC called the most matching CNVs, PennCNV-Affy ranked second. In the non-overlapping group, GTC called the least CNVs. With regards to the reproducibility of CNV calling, larger CNVs were usually replicated better. PennCNV-Affy shows the best consistency while Birdsuite shows the poorest.

**Conclusion:**

We found that PennCNV outperformed the other three packages in the sensitivity and specificity of CNV calling. Obviously, each calling method had its own limitations and advantages for different data analysis. Therefore, the optimized calling methods might be identified using multiple algorithms to evaluate the concordance and discordance of SNP array-based CNV calling.

## Background

Copy number variation (CNV) is a type of genetic variation that is widely found in human and other mammalian genomes. It includes genomic deletion, duplication, and complex rearrangement that range from 100 base pairs to several mega base pairs in size [1]. A substantial number of CNVs have significant impact on complex human diseases, such as cancer [2], autism [3], and even susceptibility to HIV [4], due to the fact that they can disrupt gene structure and affect gene regulation [1]. Therefore, studies on CNVs can further our understanding of the genetic etiology of human diseases.

To date, approximately 180,000 CNVs have been reported in the Database of Genomic Variants (DGV, see URLs). Arising from the completion of the Human Genome Project and the HapMap Project, a large number of genetic variations associated with human phenotypes or complex diseases have been identified by SNP-based genome-wide association studies (GWAS) [5]. CNVs might assist us in finding the missing heritability in GWAS. There are many methods available for CNV detection, such as microarray and Polymerase Chain Reaction (PCR) based technologies [6]. SNP array and array-based comparative genomic hybridization (CGH) are two of the most frequently used high throughput platforms. The Affymetrix (Santa Clara, CA, USA) Genome-Wide Human SNP Array 6.0 contains more than 1,800,000 probes, including 906,600 probes to detect SNPs and 946,000 probes to detect structural variations. The Agilent 1 M CGH Array contains approximately 1 million 60-mer oligonucleotide probes for CNV detection. It remains unclear whether the widely used SNP-array-based CNV calling methods can provide sufficient concordance with CGH in CNV detection.

The objective of this paper was to evaluate the performances of publicly available software packages that are used to call CNVs from SNP arrays. The CGH-based CNV detection results derived from 20 HapMap samples were used as a “gold standard” due to their high Signal-to-Noise Ratio and detection accuracy, which were described in detail by Park et al. [7]. Nowadays, the same 20 HapMap samples have also been studied using SNP 6.0 arrays in the Phase II HapMap Project [8].

## Availability and requirements

**Project name**: Comparison of four software packages in CNV calling

**Operating system(s**): XP 64bit Windows PC server

**Web Server**: Apache 2.2.4

**Programming language**: PHP 5.2.1& MySQL 5.0.27

**Other requirements**: ZendOptimizer 3.2.0, phpMyAdmin4.1.4

Scripting software—Edit plus

In order to evaluate four CNV detecting software packages specifically developed for the Affymetrix 6.0 SNP array platform, we should firstly finish parameters setting for each software package. Only when following their manuals could we start and continue the software packages, we then follow the manual issued on the official website of each software to finish the default settings (it was recommended and necessary). For example, we made all twenty-six SNP 6.0 array datasets pass the Quality Control (QC) threshold of the software package GTC as a default setting. And since dChip had no parameters setting program, we then used the output information of the genotyping results of GTC as the input data for subsequent analyses for it [9]. This was exactly instructed by the manual of dChip. For Birdsuite and PennCNV-Affy, CNV detection was performed according to the manuals on their official websites with default settings.

After parameters setting for each software package, we also set the software running environment. The samples that passed GTC QC were selected as the baseline for the SNP array settings. The results obtained by performing the “Normalize & Model” function were used for the subsequent CNV calling. And the CNV calling should in the following environment settings: 1) HMM was used as the algorithm; 2) 20% of the samples were trimmed; 3) the CNV step-width was set to 0.5.

## Implementation

We selected a published CNV dataset generated by CGH with a resolution of 24 million probes per sample as the “gold standard ” to evaluate four CNV detecting software packages developed for the Affymetrix 6.0 SNP array platform, including GTC (version 4.0), Birdsuite (version 1.5.3), dChip (version 2/25/2009), and PennCNV-Affy (version 11/21/2008).

**Figure 1:**
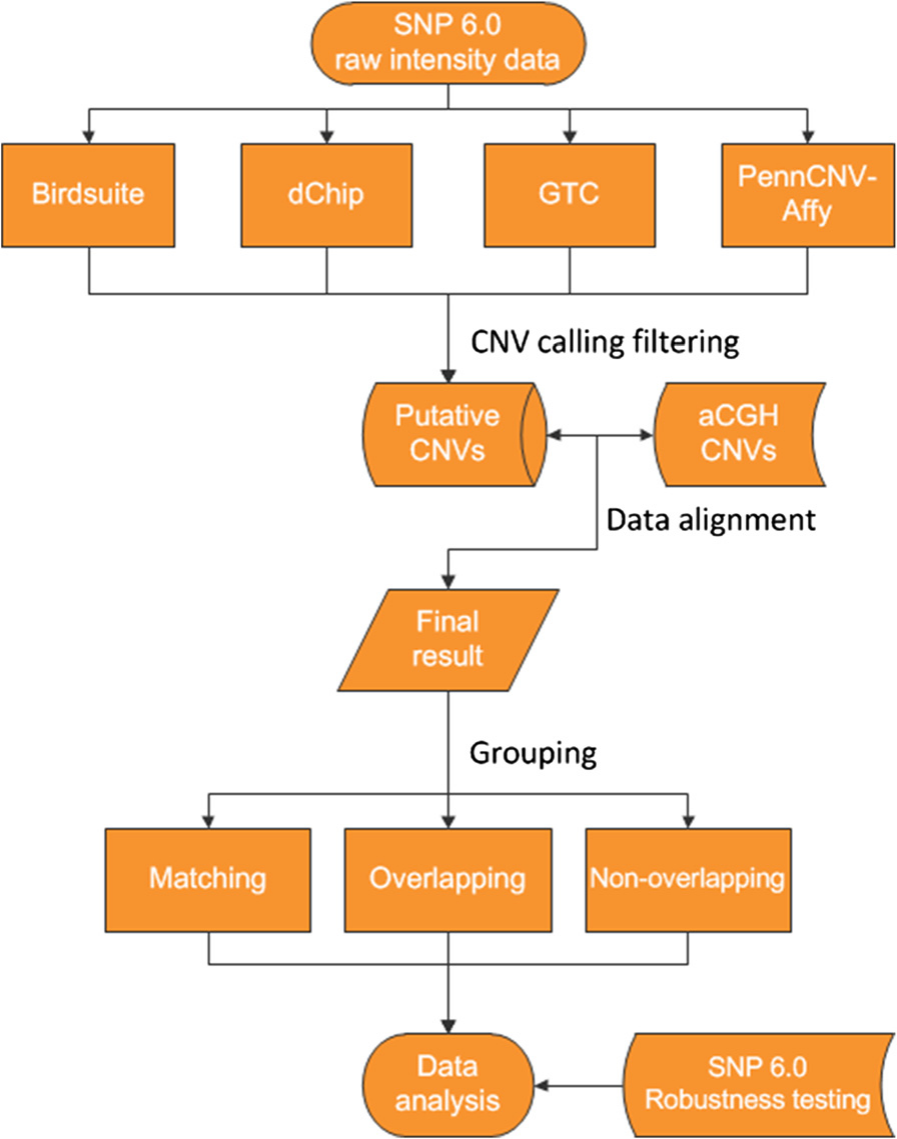
Flowchart of the study.

Compared with the CGH-based dataset, the CNVs identified by the four software packages were then divided into three groups—a matching group, an overlapping group and a non-overlapping group. The success rate, average stability rate, sensitivity, consistence and reproducibility of these four software packages were assessed compared with the “gold standard”. Specially, we also compared the efficiency of detecting CNVs simultaneously by two, three and all of the software packages with that by a single software package (Figure 1).

### Datasets

The CGH-based CNV data for the 20 HapMap samples (10 CHB and 10 JPT individuals) were obtained from Park et al. [7]. The original genotype data for the same 20 individuals based on the SNP 6.0 array were downloaded from the HapMap Project website. Another three DNA samples from the Chinese population were used twice on the SNP 6.0 array platform for reproducibility evaluation of the software packages. In total, 46 datasets for 23 individuals were involved in this study.

### CNV calling

GTC, dChip and PennCNV-Affy, these three software packages could call CNVs step by step according to their manuals. What worth noticing was that Birdsuite provided three algorithms instead of one, but only Canary and Birdseye algorithms produced CNV information. So especially, the filtration criteria for the obtained raw data was necessary, and it should included the following points: 1) a corresponding confidence value < 0.1 for Canary and a lod value > 5; 2) a marker value ≥ 3; 3) a fragment length ≥ 1 kb for Birdseye [10]. A combination of the results from the Canary and Birdseye algorithms was considered to be the final output of Birdsuite. Generally, we preferred selecting the longer one as the final result when the boundaries of the CNVs were not consistent with each other.

### Comparison methods

Our results suggested that currently available microarray platforms were complementary, and the number and type of CNVs detected might be diverse due to different microarray probe distributions, sample labeling and hybridization chemistries and algorithms [11]. For instance, through comparing the CNVs detected by four software packages, we found that the CGH was sensitive to detecting small (<30 kb) CNVs while SNP array-based CNV calling algorithms often missed them even though the probe coverage on the SNP array were sufficient on these loci. So we ignored CNVs that were smaller than 30 kb in size for better comparability between the two platforms. Then the downloaded raw data of the SNP 6.0 array from the HapMap Project were respectively analyzed by Birdsuite, dChip, GTC and PennCNV-Affy to obtain putative CNVs. These detected CNVs were then compared with the CNVs identified by the CGH platform reported by Park et al. [7]. The final results were divided into three groups: a matching group, in which CNVs exhibited ≥ 50% reciprocal overlap between the two platforms; a non-overlapping group, in which CNVs did not have any overlap; and the overlapping group, which included all of the remaining CNVs.

The total CNVs number, the mean and median sizes, and the distribution of the three groups of CNVs were thoroughly investigated to evaluate the success rate, the detecting bias, the sensitivity and the reproducibility of the four software packages designed for the SNP 6.0 platform in the following sections [12].

## Results and discussion

### Overview of CNVs detected by SNP array-based algorithms and CGH

First of all, a list of the mean size and median size of the CNVs detected by the four software packages from the SNP array and the 11,759 CNVs reported by CGH platform was shown in Table 1. We found that CGH was sensitive to detecting small (<30 kb) CNVs, which were often missed by SNP array-based CNV calling algorithms (Additional file 1 and Table 1). For better comparability between CGH and SNP6.0, we focused only on the detection of CNVs that were larger than or equal to 30 kb in size. Birdsuite called the largest number of CNVs (951); dChip called 639 CNVs, PennCNV-Affy called 564 of them and GTC called 205. The CNVs called by Birdsuite, dChip, and PennCNV-Affy had approximately the same mean (≈150 kb) and median (≈80 kb) in sizes. However, the mean and median sizes of the CNVs called by GTC were more than two times larger than those called by the other three software packages. Though the comparison of the mean size and median size of the CNVs detected by the four software packages, we initially concluded that GTC had certain bias in calling CNVs while the other three software packages had not. Further were discussed below.

**Table 1:** Summary statistics of CNVs called by four software packages

**Software or platform**	**Mean size (bp)**	**Median size (bp)**	**Total amount**	**Success rate**
Birdsuite	127,235	76,082	951	8.1%
dChip	178,920	83,144	639	5.4%
GTC	316,932	181,000	205	1.7%
PennCNV-Affy	152,634	87,813	564	4.8%
CGH&	19,040	11,502	11,759	100%

&The data are from Park *et al.*[12].

Secondly, the grouping of the CNVs detected by the four software packages was discussed in Table 2. Compared with the CNVs reported by CGH, GTC detected the most matching CNVs (66.3%), and PennCNV-Affy detected a fairly high number (45.9%); Birdsuite and dChip detected 41.3% and 9.4% matching CNVs respectively, which were obviously lower than the results of GTC or PennCNV-Affy. In the non-overlapping group, the software package that detected the smallest proportion of non-overlapping CNVs was GTC (only 29.8%); and the largest proportion was by PennCNV-Affy which reached 40.3%. For the overlapped CNV group, dChip detected the most CNVs (31.0%) and GTC detected the least CNVs (3.9%). The grouping of the CNVs detected by the four software packages showed the overlap ratio of them. Generally speaking, moderate overlap ratio between the two platforms indicated that such kind of software package was suitable for the detection of both the known CNVs and the unknown CNVs. In our study, PennCNV-Affy and Birdsuite could meet such standard and the former one performed better.

**Table 2:** Comparison of CNVs between two high-throughput platforms

**Software**	**Matching group**	**Overlapping group**	**Non-overlapping group**
Birdsuite	41.3%	12.4%	46.3%
dChip	9.4%	31.0%	59.6%
GTC	66.3%	3.9%	29.8%
PennCNV-Affy	45.9%	13.8%	40.3%

**Figure 2:**
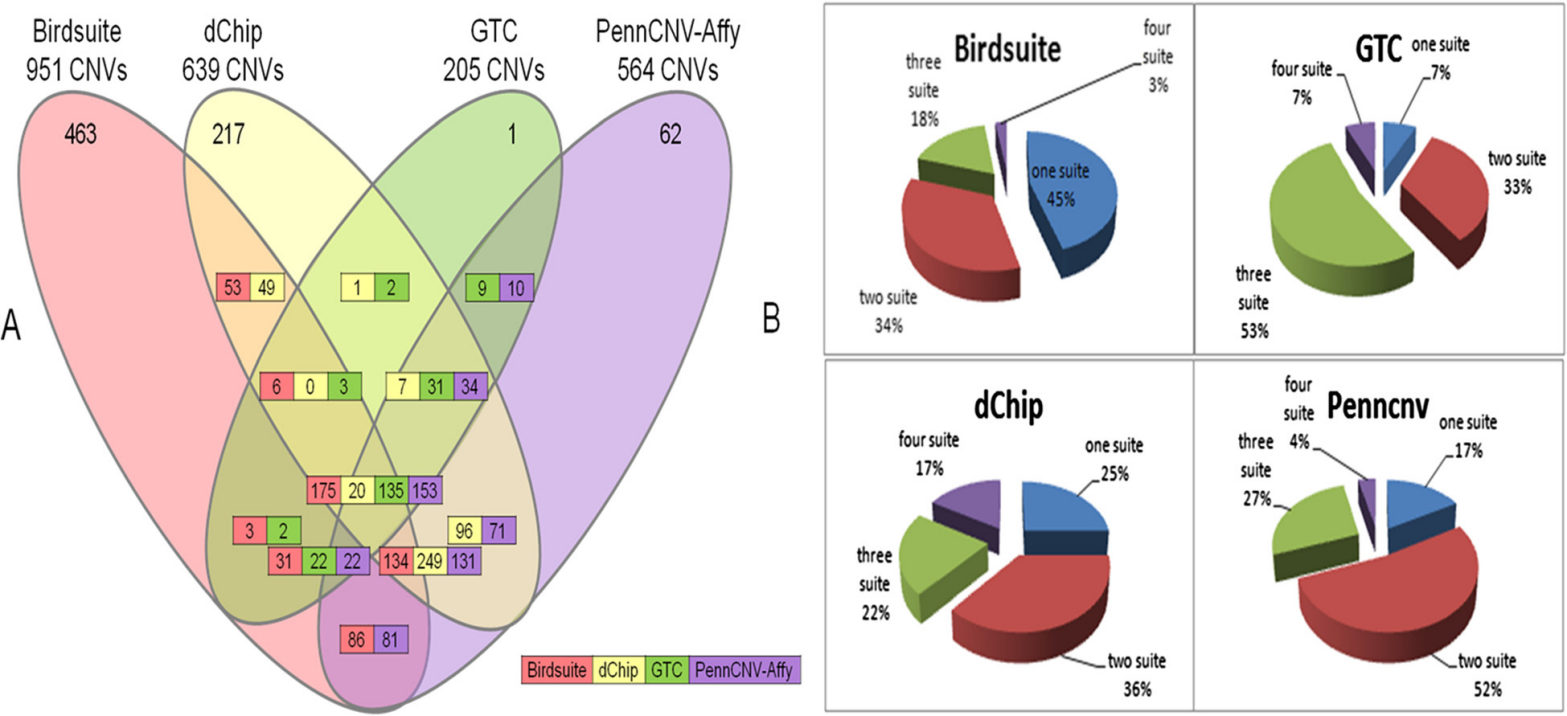
**Study of CNV calling in matched groups. (A)** Venn showing CNV calls generated by four software packages **(B)** CNV calls generated by multiple software packages.

Figure 2 illustrates a comprehensive comparison of CNVs detected by the four software packages. In Figure 2A, the four colors represented the CNVs that were called from four software packages (red for Birdsuite, yellow for dChip, green for GTC and purple for PennCNV-Affy). The numbers of CNVs in the colored rectangles indicated the amount of overlapped CNVs called by two, three or four software packages. Overlapping CNVs referred to those who had at least 1 bp but less than 50% of overlapping bp shared by two CNVs. Thus, multiple numbers were also generated for overlapped CNVs in two or three software comparisons. Two numbers in one rectangle indicated overlapped CNVs in two software packages and three numbers indicated overlapped CNVs in three software packages. We found that each software’s ability in calling CNVs was not all-powerful and had specific advantages and limitations. We therefore proposed that the combined use of multiple software packages could provide us with higher accuracy and reliability as shown in Figure 2B. In Figure 2B, the entire pie referred to the total quantity of the matching CNVs of each software package. “one suite” meant the percentage of the amount that were detected by itself alone, and “two suite” meant the percentage of the amount that were detected by it and one other software package, and so on. Obviously, a large fraction of the matching CNVs were detected by several software packages simultaneously. Also, if we used only one method, the calling effect would not certainly be so good as the combined use of multiple software packages. Of course, it was not true that the more methods we used, the better the CNV calling effect we would get (Figure 2B). The choice of calling methods mostly depended on our actual needs.

### Performance test

We investigated the CNVs detected by each of the four tested software packages and compared with those reported by the gold standard (CGH array). The success rate referred to the percentage of the matching and overlapping CNVs called by the tested software packages with the size more than 30 kb versus total CNVs from CGH array in 20 HapMap samples.

**Figure 3:**
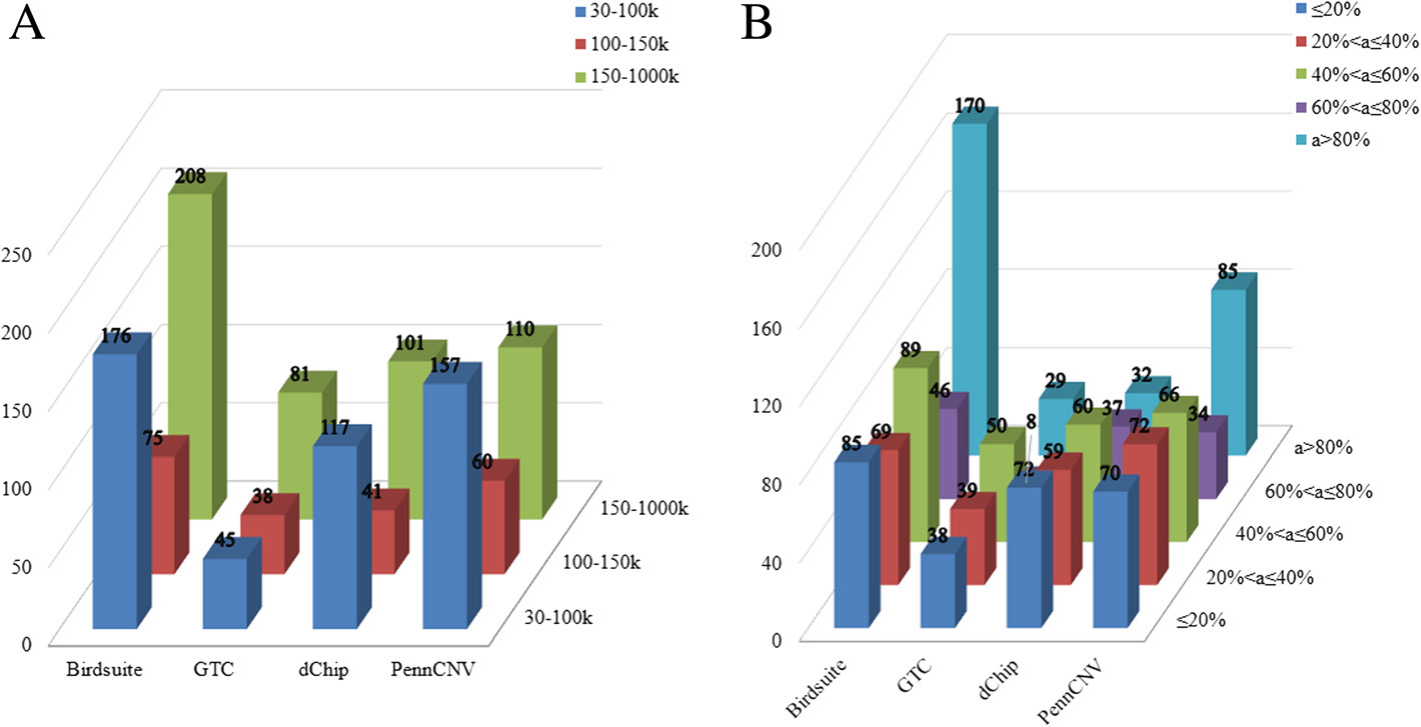
**Study Performance of CNV calling. (A)** CNV calls of size distribution. **(B)** CNV frequency of occurrence.

For one software package, notably, the success rate increased with the enlarged CNV size (Table 3). For example, the highest success rate for CNVs >150 kb was 62.3%, which was detected by Birdsuite, and PennCNV ranked secondly. Meanwhile, concerning the total CNVs number, it was easier for four software packages to call CNVs with the size distributed extremely in two tails (big or small) (Figure 3A). Such kind of bias inevitably affects our CNV calling result especially for the unknown CNVs. Although Birdsuit called the largest amount of CNV, its bias was obvious. And GTC seemed inferior because of the less total CNV number and the notably high mean and median sizes of the detected CNVs. As for dChip, the significant bias of the results was due to a big fraction of the detected CNVs with the size smaller than 30 kb, which was not within the scope of our study. The frequency of CNVs detected by PennCNV in all samples distributed most closely to the average one (Figure 3B). So we drew a conclusion that PennCNV outperformed the other three in the success rate and bias comparison (Figure 3A).

**Table 3:** The average success rate of the four CNV-calling methods, according to CNV length and frequency (a)

**CNV length**	**Total amount of CNV**	**Amount of CNVs called by birdsuite**	**Amount of CNVs called by GTC**	**Amount of CNVs called by dChip**	**Amount of CNVs called by PennCNV-Affy**
30-100K	1075	176(16.4%)	45(4.2%)	117(10.9%)	157(14.6%)
100-150K	209	75(35.9%)	38(18.2%)	41(19.6%)	60(28.7%)
150-1000K	334	208(62.3%)	81(24.3%)	101(30.2%)	110(32.9%)
**CNV frequency**					
≤20%	417	85(20.4%)	38(9.1%)	72(17.3%)	70(16.8%)
20%<a<=40%	216	69(31.9%)	39(8817.8%)	59(27.3%)	72(33.3%)
40%<a<=60%	188	89(47.3%)	50(26.6%)	60(31.9%)	66(35.1%)
60%<a<=80%	107	46(43%)	8(7.5%)	37(34.6%)	34(31.8%)
a>80%	699	170(24.3%)	29(4.1%)	32(4.6%)	85(12.2%)

### Consistency of the total quantity of the detected CNVs

The CNVs in the matching group, which were simultaneously detected by two, three or all of the four software packages, had a large bias when compared with the CNVs detected by a single software package alone (Figure 2A).

The matching group showed an overall homogeneity of the called CNVs for all of the types of software packages (Figure 2B). Thus, the observation that the consistency of the detected CNVs between the SNP array and CGH array was preferentially limited in the overlapped region rather than in the specific region suggested a possible signal instability and unreliable specific detection due to the SNP array.

### Size and chromosome distribution of CNV

**Figure 4:**
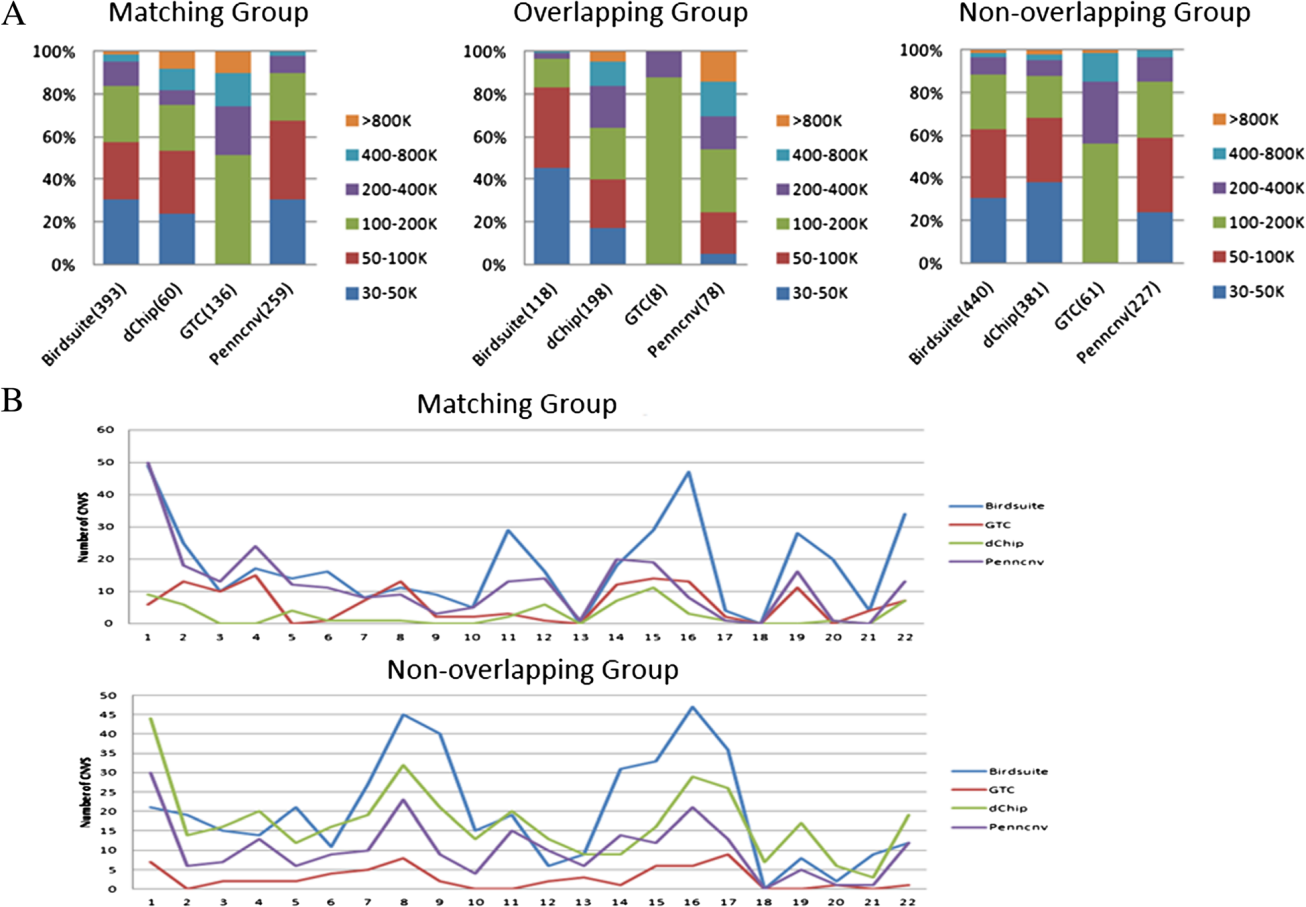
**Study CNV calling of size distribution and Chromosome distribution. (A)** CNV calls of size distribution **(B)** CNV calls of Chromosome distribution.

The size distributions of the grouped CNVs for all four software packages were shown in Figure 4. In Figure 4A, each bin represented a different range of CNV lengths and the bars showed the percentage of CNVs in each size bin. The numbers in the parentheses indicated the total number of CNVs in each column (i.e., the total number of CNVs called by each program). Figure 4A illustrated the sizes of the detection results of comparing CGH-based CNV calling and SNP 6.0-based CNV calling in a total of 20 HapMap samples by four software packages.

As described previously, we focused only on the CNVs of ≥ 30 kb in size that were reported by the CGH platform [7]. Three groups of CNVs were analyzed separately (Table 2), including matching, overlapping and non-overlapping CNVs.

In Figure 4B, each broken line represented the chromosome distribution of the CNV called by different software packages, and the inflection points showed the number of CNVs in each chromosome. Figure 4B illustrated the chromosome distribution of the detection results of comparing CGH-based CNV calling and SNP 6.0-based CNV calling in a total of 20 HapMap samples by four software packages.

In the matching group, the matching CNVs from Birdsuite were distributed mainly on chromosomes 1 and 16 (Figure 4B). Chromosome 1 also contained peaks of CNVs from dChip and PennCNV-Affy. Relatively fewer CNVs on chromosomes 9, 10, 13, 18, 20 and 21 were called by almost all of the tested four software packages. Due to the small number of total matching CNVs from GTC, relatively fewer GTC-called CNVs were observed across all of the chromosomes, with notably less on chromosomes 5, 10, 11, 12, 13, 18, and 20. In the non-overlapping group, there was a limited proportion of CNVs distributed on the chromosome. Only non-overlapping CNVs from Birdsuite formed two peaks on chromosome 9 and 17, and the other three software packages formed CNV peaks on chromosomes 8 and 17.

Additionally, many of the matching CNVs were on chromosome 1, except the detection results of GTC. One possible explanation for this distribution was that chromosome 1 had the largest number of genes. Chromosome 18 contained the fewest detected CNVs, even compared with the detected CNVs on other shorter chromosomes. Interestingly, the most CNVs identified by Birdsuite were distributed on chromosome 1 and chromosome 16. Although chromosome 13 was longer than chromosome 16, very few CNVs were distributed on chromosome 13. Specifically, data for chromosome X and Y were not shown because PennCNV-Affy didn’t carry sex chromosome information.

### Reproducibility test

To analyze the reproducibility of CNV calling for each of the four tested software packages, we performed the same experiment twice on a SNP 6.0 array using three Chinese DNA samples. The CNVs were considered to be replicated when at least 50% of the sequences in both CNVs overlapped.

Table 4 showed that there were certain defects in the reproducibility of the data generated by the four software packages, and the sensitivity of the defects to CNVs of different lengths were different, including the overlapping (equal or more than 1 bp) proportions of all of the CNVs and that of the CNVs larger than 15 kb and the CNVs larger than 30 kb.

**Table 4:** Batch effect test

	**All (%)**	**>15K (%)**	**>30k (%)**
Birdsuite	41.7	51.6	52.9
GTC	52.9	52.9	52.9
dChip	56.7	73.0	75.0
PennCNV-Affy	88.6	85.0	85.7

Our results demonstrated that larger CNVs were better replicated, and only GTC performed consistently in the duplicated experiments. Comparing the four software packages, PennCNV-Affy shown the highest consistency and Birdsuite the poorest, which were similar to the results reported by Zhang et al. [12].

### Analysis of the non-overlapping group

**Figure 5:**
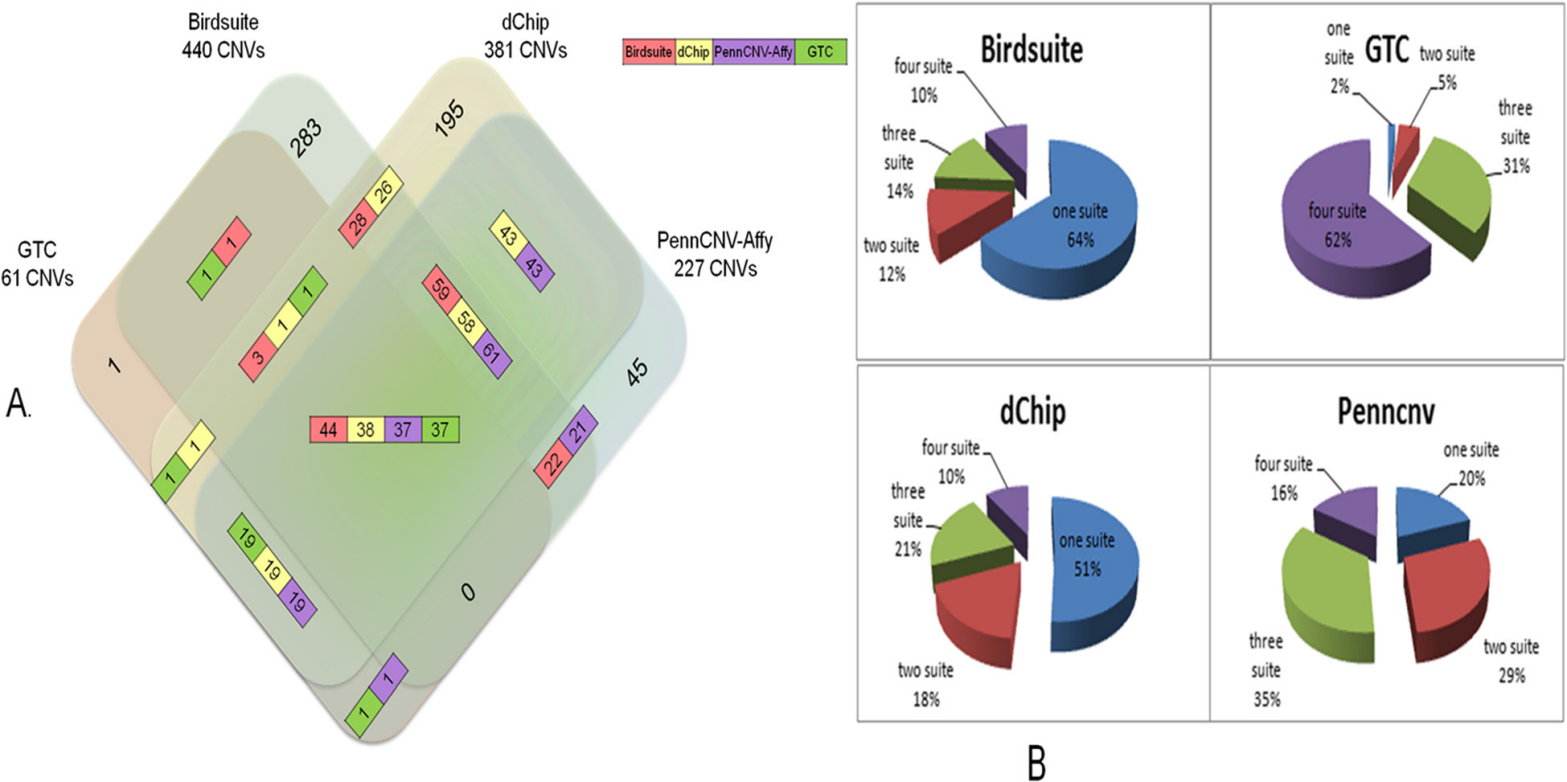
**Study CNV calling in non_overlap group. (A)** Venn showing CNV calls generated by four software packages **(B)** CNV calls of multiple software packages.

**Figure 6:**
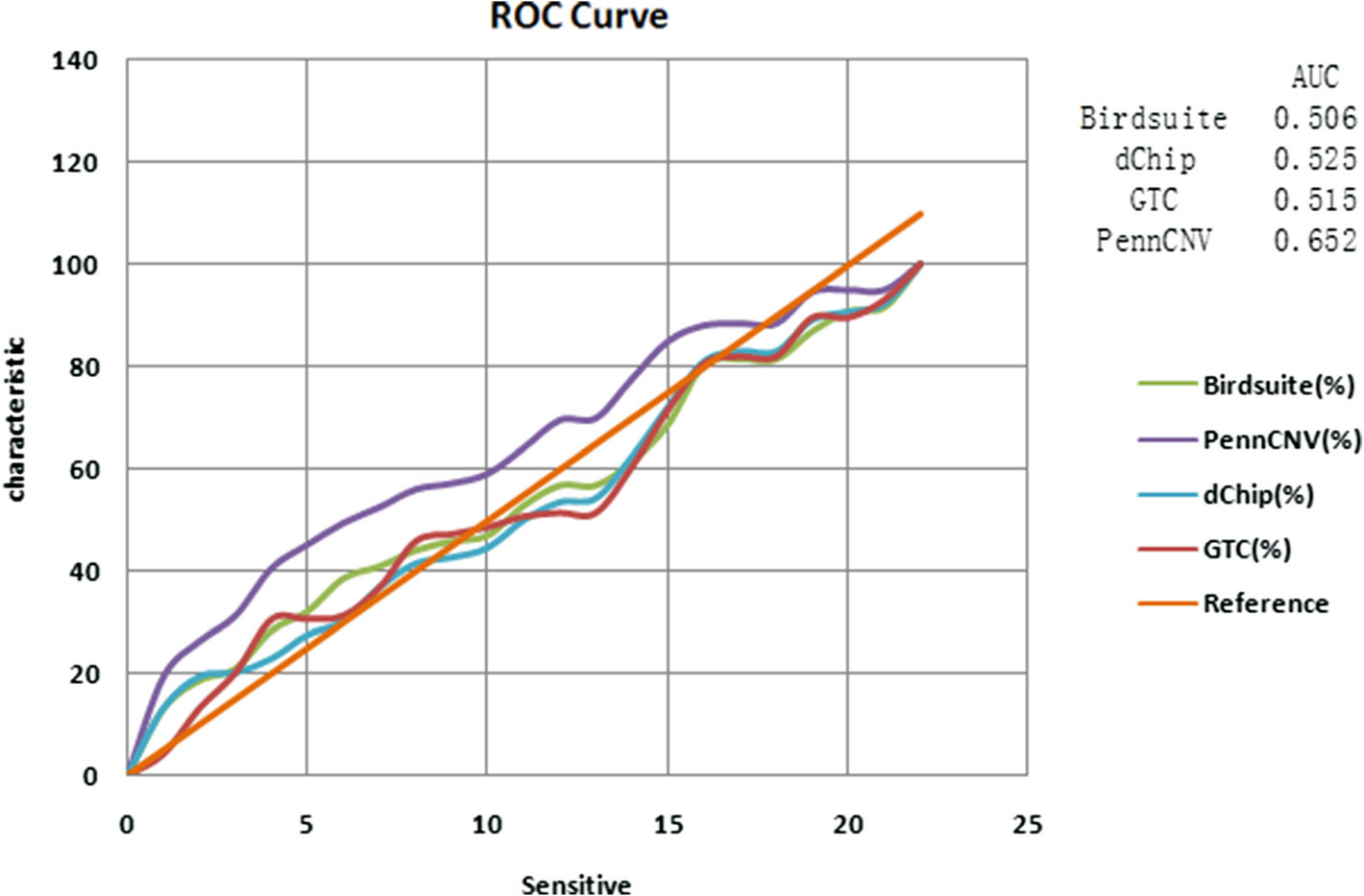
ROC/AUC of study.

In this four-dimensional Venn diagram (Figure 5A), for the non-overlapping group, the total number of CNVs detected by Birdsuite, GTC, dChip and PennCNV were 440, 61, 381 and 227, which could be considered to be false-positive CNVs after comparison with the CGH data. The remainder of the 283/1/195/45 CNVs in the non-overlapping group were those that could be detected by only one of SNP-array softwares or CGH-array software (Additional file 2). We used a Receiver Operating Characteristic (ROC) curve to graphically represent the false negative and false positive rates of each of the four tested software packages. Statistically, more area under the curve meant that the method could identify more true positive results while minimizing the percentage of false positive results. Accordingly, the AUC (Area under ROC Curve) of the four mentioned software packages were as follows: 0.506 for Birdsuite, 0.525 for dChip, 0.515 for GTC, and 0.652 for PennCNV (Figure 6), which indicated that PennCNV outperformed the other three packages, and the performances of the other three software packages were similar.

The percentage of CNVs simultaneously called by the different combinations of other software packages was indicated in the pie charts (Figure 5B). Most CNVs called by GTC could be validated by the other software packages, whereas most CNVs called by Birdsuite and dChip could be validated only by themselves.

## Conclusions

The objective of this research was to investigate the publicly available software algorithms for CNV calling from raw data produced by the Affymetrix 6.0 SNP array platform. For this purpose, four software packages, e.g., Birdsuite, dChip, GTC and PennCNV-Affy were evaluated. In our study, the total quantity, the mean and median sizes and the grouping of the detected CNVs were thoroughly investigated to evaluate the success rate, the detecting bias, the sensitivity and the reproducibility etc. of the four software packages.

Through our study, we found that PennCVN-Affy outperformed the other three software packages on the whole. First of all, the parameters setting for PennCNV-Affy could be very easy. It were performed according to the manual on its official websites with default settings which could save time and make it general.

Secondly, PennCNV performed better than the other three ones in the success rate and bias comparison. Although the total quantity of CNVs called by PennCNV was not the largest among these four software packages, PennCNV showed less bias when calling CNVs, which enabled it to find similar amount of both known and unknown CNVs. We considered this kind of balance also reflected its high sensitivity and high specificity.

In addition, we used a Receiver Operating Characteristic (ROC) curve to graphically represent the false negative and false positive rates of each of the four tested software packages. Statistically, PennCNV outperformed the other three packages, and the performances of the other three software packages were similar.

Moreover, in the reproducibility test, CNVs were categorized into three groups, including all CNVs, CNVs larger than 15 kb, and CNVs larger than 30 kb. GTC shown no differences among these groups because all of the CNVs called by GTC were larger than 30 kb. Birdsuite, dChip, and GTC had only an approximately 55% consistency if the CNVs shorter than 30 kb were considered. However, PennCNV obtained a consistent CNV calling as high as 87% even if it used the same algorithm with dChip and GTC, which indicated that only 13% of the CNVs called by PennCNV were not found in the CGH-based CNV dataset.

Based on the above reasons, PennCNV seemed to be a reasonable and acceptable option when choosing single software package for CNV detection. But it was worth noting that the algorithms themselves might cause differences in the CNV detection [1]. Meanwhile, software packages also have different emphases when they employed different algorithms. For example, Birdsuite had a higher success rate but lower reproducibility. In contrast, GTC obtained high specificity but lower sensitivity and appears to be more conservative than other types of software.

Obviously, a large part of the matching CNVs were detected by several software packages by the same time. Also, if we used only one method, the calling effect would be certainly not so good as the combined use of multiple software packages. Besides, the concordance between the SNP 6.0 and CGH platforms was much lower than 40%, and the different algorithms of each software packages would also make the detecting result diverse.

Therefore, we proposed the combined use of multiple software packages, thus could provide us higher accuracy and reliability in the CNV detecting.

### URLs

The Hapmap Project website, http://hapmap.ncbi.nlm.nih.gov/;

The official PennCNV website, http://www.openbioinformatics.org/penncnv;

Database of Genomic Variants (DGV), http://dgvbeta.tcag.ca/dgv/app/home

UCSC Genome Bioinformatics Site, http://genome.ucsc.edu/

## Competing interests

The authors declare that they have no competing interests.

## Authors’ contributions

XZ performed the CNV calling experiment and statistical analysis, and drafted the manuscript. RD participated in the statistical analysis and manuscript preparing. SL carried out the SNP array experiment. HW, LJ and FZ conceived the study. All authors read and approved the final manuscript.

## Supplementary Material

Additional file 1 CNV calls for 20 Hapmap samples by 4 software packages. Click here for file

Additional file 2 Comparison of CNV calls of 4 software packages. Click here for file
